# Real-world evidence of baseline soluble CD25 as a prognostic biomarker and indicator of differential EGFR–TKI benefit in stage IV lung adenocarcinoma

**DOI:** 10.3389/fonc.2026.1810131

**Published:** 2026-07-10

**Authors:** Huiru Guo, Lingshuang Liu, Veronika Lindberg, Yingjun Xue, Jan P. A. Baak

**Affiliations:** 1 Department of Medical Oncology VI, Longhua University Hospital, Shanghai, China; 2 Department of Statistics, Lintech AS, Kristiansand, Norway; 3 Department of Laboratory Medicine, Longhua University Hospital, Shanghai, China; 4 Department of Molecular Digital Pathology, Stavanger University Hospital, Stavanger, Norway

**Keywords:** antiangiogenic therapy, biomarker, EGFR tyrosine kinase inhibitors, immune checkpoint inhibitors, lung adenocarcinoma, prognosis, soluble CD25, treatment response

## Abstract

**Background:**

Outcomes in stage IV lung adenocarcinoma (LUAD) remain heterogeneous despite established genomic and immune biomarkers. We evaluated the prognostic and predictive value of soluble interleukin-2 receptor (sCD25), a marker of systemic inflammation and immune regulation.

**Methods:**

In this prospective observational real-world study (2020–2024), 133 consecutive patients with newly diagnosed stage IV LUAD, ECOG performance status 0–1, and survival ≥3 months were enrolled. Overall survival (OS) was the primary endpoint. Associations between sCD25 and OS were assessed using Kaplan–Meier estimates, multivariable Cox regression, and propensity score matching. Treatment-by-biomarker interaction analyses evaluated EGFR tyrosine kinase inhibitors (TKIs), immune checkpoint inhibitors (ICIs), and antiangiogenic therapy.

**Results:**

In the whole group, at a median follow-up of 24.1 months, 52 deaths occurred during follow-up. Elevated sCD25 (≥441 U/mL) was associated with shorter OS (hazard ratio [HR], 5.70; 95% CI, 2.94–11.07; P <.0001) and remained prognostic in multivariable analysis (HR, 4.23; 95% CI, 2.01–8.91; P <.001) and after propensity score matching (HR, 4.61; 95% CI, 2.01–10.62; P <.001). One- and three-year OS rates were 97% and 78% for low sCD25 versus 68% and 27% for high sCD25. A significant treatment interaction was observed for EGFR–TKIs, with benefit limited to the high-sCD25 subgroup (HR, 0.41; 95% CI, 0.19–0.88; P = .02). No significant interactions were observed for ICIs or antiangiogenic therapy.

**Conclusion:**

Baseline sCD25 is a biomarker in stage IV LUAD —strongly prognostic and suggests differential benefit from EGFR–TKIs—and may help refine EGFR–TKI selection. Multicenter validation is warranted.

## Graphical Abstract

**Figure:**
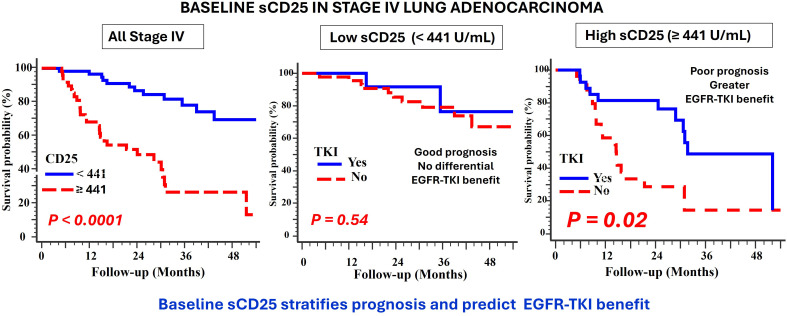
Baseline soluble CD25 (sCD25) levels were evaluated in 133 patients with stage IV lung adenocarcinoma (ECOG performance status 0–1, ≥3 months survival). Patients were stratified by sCD25 levels into low (<441 U/mL) and high (≥441 U/mL) groups. Low sCD25 levels were associated with favorable prognosis and showed no clear association with benefit from EGFR–tyrosine kinase inhibitors (EGFR–TKIs). Interpretation is limited by the small number of events. In contrast, high sCD25 levels were associated with poorer prognosis but greater benefit from EGFR–TKI therapy. Overall, baseline sCD25 correlates with overall survival and may serve as a biomarker to identify patients with differential response to EGFR–TKI treatment.

## Introduction

Lung cancer remains the leading cause of cancer-related mortality worldwide, with most patients presenting with stage IV disease at diagnosis. Lung adenocarcinoma (LUAD) is the most common histologic subtype (1, 2). Despite advances in genomic testing and immune profiling, clinical outcomes in advanced LUAD remain highly variable.

Previous biomarker studies in advanced non–small-cell lung cancer (NSCLC) have been limited by heterogeneity in stage, histology, and performance status, as well as small sample sizes and short follow-up (3). In addition, inclusion of patients with very short survival (<3 months)—who rarely complete systemic therapy—may complicate interpretation of biomarker performance among longer-term survivors (4). Although genomic drivers such as EGFR, ALK, KRAS, MET, RET, ROS1, and BRAF, as well as immune biomarkers such as PD-L1 and tumor mutational burden (TMB), guide therapy, substantial outcome variability persists (5). This highlights the need for accessible biomarkers that capture the interplay between tumor biology, host immunity, and treatment response.

Soluble interleukin-2 receptor (sCD25; sIL-2Rα) reflects IL-2 pathway activation and T-cell regulation (6–9). Elevated sCD25 has been associated with tumor burden, inferior survival in NSCLC, and reduced benefit from immune checkpoint inhibitors (ICIs) (10–13). However, its prognostic and predictive significance specifically in stage IV LUAD has not been established due to methodological limitations in prior studies.

Serum ferritin, although a marker of iron stores, also increases in states of systemic inflammation and tumor-driven immune dysregulation. Elevated ferritin has been associated with poor prognosis in NSCLC and other cancers (14–17), but has not been validated in homogeneous stage IV LUAD cohorts, making it a relevant comparator.

In this prospective study, we evaluated whether baseline sCD25 independently predicts survival and treatment benefit in patients with stage IV LUAD, ECOG performance status 0–1, and survival ≥3 months, with ferritin assessed as a secondary comparator biomarker.

## Methods

### Study design and patients

This prospective, noninterventional observational cohort study was conducted at Longhua University Hospital (LUH), Shanghai, China. The protocol was approved by the LUH Institutional Review Board (IRB 2022LCSY024) and conducted in accordance with international ethical standards, including the Declaration of Helsinki and CIOMS guidelines (18, 19). Written informed consent was obtained from all participants.

Consecutive patients with pathologically confirmed stage IV NSCLC were screened between January 1, 2020, and December 31, 2024.

Inclusion criteria were:

1. adenocarcinoma histology;
2. stage IV disease according to the eighth edition TNM classification;
3. ECOG performance status (PS) 0–1;
4. availability of baseline blood samples; and
5. survival ≥3 months after diagnosis.

Exclusion criteria were:

- ECOG PS 2–4;
- non-adenocarcinoma histology;
- incomplete baseline laboratory data (including missing sCD25 measurement);
- loss to follow-up; and
- survival <3 months after diagnosis.

The restriction to patients with survival ≥3 months was a predefined design choice to ensure inclusion of individuals with sufficient follow-up and opportunity to receive systemic therapy.

Of 385 screened patients, 133 consecutive cases met all eligibility criteria (**Supplementary Figure 1**).

### Data collection and follow-up

Baseline demographics, TNM stage (IVA vs IVB), treatment exposures, and laboratory biomarkers were obtained from electronic medical records. Follow-up was performed monthly through clinic visits or structured telephone contact until March 31, 2025.

### Treatments

Treatments included platinum-based chemotherapy (20), EGFR tyrosine kinase inhibitors (TKIs), immune checkpoint inhibitors (ICIs), and bevacizumab-based antiangiogenic therapy. Radiotherapy was delivered as clinically indicated (21). Treatment variables reflect exposure at any time during the disease course; patients frequently received sequential or combination therapies, including chemotherapy and/or radiotherapy in addition to targeted or immune-based treatments. Additional treatment details are provided in the **Supplementary file**.

### Baseline variables and laboratory methods

Baseline variables included age, sex, TNM stage (22), EGFR mutation status (EGFR, other driver mutations, or none), body mass index (BMI) (23, 24), presence of type 2 diabetes mellitus, and ferritin. ECOG PS was not included in regression models because all enrolled patients had ECOG 0–1.

Therapy choice was not included as a confounder in baseline sCD25 analyses because treatment is determined after biomarker measurement. Sensitivity analyses evaluating treatment heterogeneity confirmed that this did not materially affect results.

Baseline blood samples were drawn at the first LUH visit. sCD25, ferritin, and (in 2020–2021 only) VEGF were quantified using standardized immunoassays; details regarding assay characteristics and coefficients of variation are provided in the **Supplementary Table S1**.

### Statistical analysis

The primary endpoint was overall survival (OS), defined from the date of pathological diagnosis to death from any cause. All analyses followed a prespecified statistical plan and adhered to STROBE reporting guidelines (**Supplementary Figure 1**).

Associations between sCD25 and OS were assessed using Kaplan–Meier analysis and Cox proportional hazards regression. Proportional hazards assumptions were formally evaluated. sCD25 was analyzed as both a continuous and a categorical variable; continuous modeling was performed to evaluate its independent prognostic effect without reliance on a specific cutoff.

A cut point of 441 U/mL was derived using receiver operating characteristic (ROC) analysis and the Youden index. To assess robustness and reduce dependence on a single data-derived threshold, exploratory analyses were additionally performed using alternative categorizations based on the median, tertiles, and quartiles of the sCD25 distribution where sample size permitted. Hazard ratios and survival discrimination remained directionally consistent across these alternative categorizations, supporting the robustness of the association between elevated sCD25 and inferior survival. Exploratory treatment-specific threshold analyses for EGFR–TKIs, immune checkpoint inhibitors, and antiangiogenic therapy were also performed; however, no alternative threshold consistently improved prognostic discrimination compared with the ROC-derived cutoff of 441 U/mL. Internal validation of the ROC-derived cutoff was performed using bootstrap resampling (1000 iterations) to estimate 95% confidence intervals, with a fixed random seed (928) to ensure reproducibility. Covariates included age, sex, TNM stage, EGFR mutation status, BMI, diabetes mellitus, and ferritin as a marker of systemic inflammation (17). Missing data were assessed for all baseline variables. Missingness in sCD25 was primarily due to logistical and preanalytical factors related to baseline sample availability at the time of initial clinical evaluation. Patients with missing EGFR mutation status were excluded from mutation-stratified and EGFR–TKI subgroup analyses.

Analyses were performed using a complete-case approach. Given the observational design and the pattern of missingness, no imputation was performed. Given the moderate sample size and the nature of the primary biomarker, multiple imputation was not undertaken, as it would require additional assumptions that are difficult to verify and could introduce further uncertainty.

Mutation status was missing in 6 patients; these cases were excluded from mutation-specific subgroup analyses but did not materially affect overall or treatment-related results. Sensitivity analyses using alternative modeling approaches yielded consistent results, supporting the robustness of the findings.

To mitigate confounding by indication, propensity scores were estimated using baseline covariates and applied using 1:1 nearest-neighbor matching with a caliper of 0.1 and robust variance estimators (25–31). Covariate balance was assessed using standardized mean differences (SMD), with values <0.10 considered indicative of adequate balance. Details on SMD calculation methods and propensity score quality assessment covariate balance results are presented in **Supplementary Table 2**. **Supplementary Table 3** shows the quality check list of the Propensity Score Analysis. Sensitivity analyses using alternative matching algorithms confirmed the stability of findings.

A *post hoc* power analysis indicated >95% power for subgroup comparisons.

## Results

### Patient characteristics

A total of 133 eligible patients with stage IV lung adenocarcinoma were included, with median follow-up was 24.1 months (range, 3.0–60.0). Fifty-two patients (39%) had died in the follow-up at the time of analysis. Baseline characteristics associated with improved survival in univariate analysis included age <68 years (P = 0.006), female sex (P = .05), and higher BMI (≥22.1 kg/m²; P = 0.002). Diabetes mellitus was not significantly associated with survival (P = .08). EGFR mutations were associated with better survival than non-EGFR or absent mutations (P = .008) (**Table 1**). Although EGFR-TKI therapy showed a strong univariate association with improved survival (P = .001) and chemotherapy showed a modest association (P = .03), these effects were susceptible to confounding by indication and duration of treatment exposure. Baseline sCD25 was not associated with treatment allocation. In univariable analyses, no significant association was observed between sCD25 level and chemotherapy (χ² = 2.41, *P* = 0.12), radiotherapy (χ² = 0.24, *P* = 0.62), or immune checkpoint inhibitors (χ² = 0.11, *P* = 0.74). These findings were consistent in multivariable analyses, in which neither chemotherapy (OR 0.52, 95% CI 0.24–1.14; *P* = 0.10), radiotherapy (OR 0.76, 95% CI 0.35–1.65; *P* = 0.49), nor immune checkpoint inhibitor treatment (OR 1.15, 95% CI 0.51–2.55; *P* = 0.74) was associated with sCD25.

**Table 1:** Univariate overall survival analysis in stage IV lung adenocarcinoma analyses included 133 patients; numbers varied for variables with missing data, and sCD25 analyses were restricted to 109 patients (42 deaths).

Variable	Events/total	Alive at last follow-up, %	Median OS, mo (IQR)	HR (95% CI)	P
Age, year					
<68	24/74	68	52.0 (32.0)	Reference	–
≥68	27/59	54	43.0 (37.0)	2.26 (1.26–4.07)	.006
Sex					
Female	16/52	69	60.0 (19.5)	Reference	–
Male	35/81	57	45.0 (23.5)	1.74 (0.99–3.07)	.05
BMI^§^					
≥22.1	14/55	75	NE*	Reference	–
<22.1	22/46	52	15.0 (33.0)	2.93 (1.47–5.83)	.002
Diabetes mellitus					
No	42/115	63	52.0 (39.0)	Reference	–
Yes	9/18	50	31.0 (17.0)	2.30 (0.92–5.77)	.08
TNM stage					
IVA	23/60	62	45.0 (30.0)	Reference	–
IVB	28/73	62	49.0 (16.0)	1.15 (0.65–2.01)	.64
Gene mutations^†^					
Absent/non-EGFR	28/56	50	30.0 (18.3)	Reference	–
EGFR	21/71	70	52.0 (21.1)	0.45 (0.25–0.81)	.008
Radiotherapy					
No	20/68	71	42.3 (19.6)	Reference	–
Yes	31/65	52	39.0 (23.5)	1.58 (0.90–2.76)	.11
Chemotherapy					
<4 cycles	15/57	74	46.8 (22.0)	Reference	–
4–6 cycles	36/76	53	39.0 (37.0)	1.90 (1.08–3.34)	.03
TKI therapy					
No	24/49	51	21.0 (15.3)	Reference	–
Any	27/84	68	52.0 (22.9)	0.36 (0.19–0.67)	.001
Antiangiogenic					
No	25/85	71	60.0 (18.8)	Reference	–
Any	26/48	46	39.0 (23.0)	1.43 (0.81–2.51)	.22
Immunotherapy					
No	31/91	66	52.0 (24.2)	Reference	–
Any	20/42	52	39.0 (32.0)	1.38 (0.76–2.51)	.29
sCD25, U/mL^‡^					
<441	14/60	77	NE*	Reference	–
≥441	28/49	43	24.0 (21.4)	5.70 (2.94–11.07)	<.0001
Ferritin, ng/mL					
<328	21/73	71	NE*	Reference	–
≥328	31/60	48	31.0 (19.6)	2.02 (1.15–3.53)	.01
VEGF§, pg/mL					
≤16.0	6/28	79	47.1 (16.7)	Reference	–
>16.0	14/20	30	17.0 (21.9)	4.50 (1.79–11.35)	<.01

^*^ Analysis restricted to patients surviving ≥3 months. HR, hazard ratio; CI, confidence interval; IQR, interquartile range; BMI, body mass index; OS = overall survival; NE, not estimable (median not reached within follow-up).

^†^ Gene mutation data missing for 6 patients.

^‡^ sCD25 data missing for 24 patients; analyses restricted to 109 patients.

^§^ BMI: data missing for 32 (24.1%) patients.

VEGF measured only in 2020–2021 (discontinued owing to high CV); data missing for 85 (63.9%) patients.

### Association of sCD25 with survival

Receiver operating characteristic (ROC) analysis identified 441 U/mL as the optimal sCD25 cutoff for discriminating survival (AUC, 0.685; sensitivity, 69.0%; specificity, 71.6%). Results were consistent using alternative thresholds derived from median, tertiles, and quartiles. Bootstrap resampling (1000 iterations) confirmed stability of the ROC-derived cutoff and the area under the curve (95% Bootstrap CI 0.563 to 0.781). Results were consistent across exploratory analyses using alternative thresholds derived from the median, tertiles, and quartiles of sCD25, with elevated sCD25 remaining associated with inferior survival across categorizations. No alternative threshold materially improved prognostic discrimination compared with the ROC-derived cutoff of 441 U/mL.

**Figure 1:**
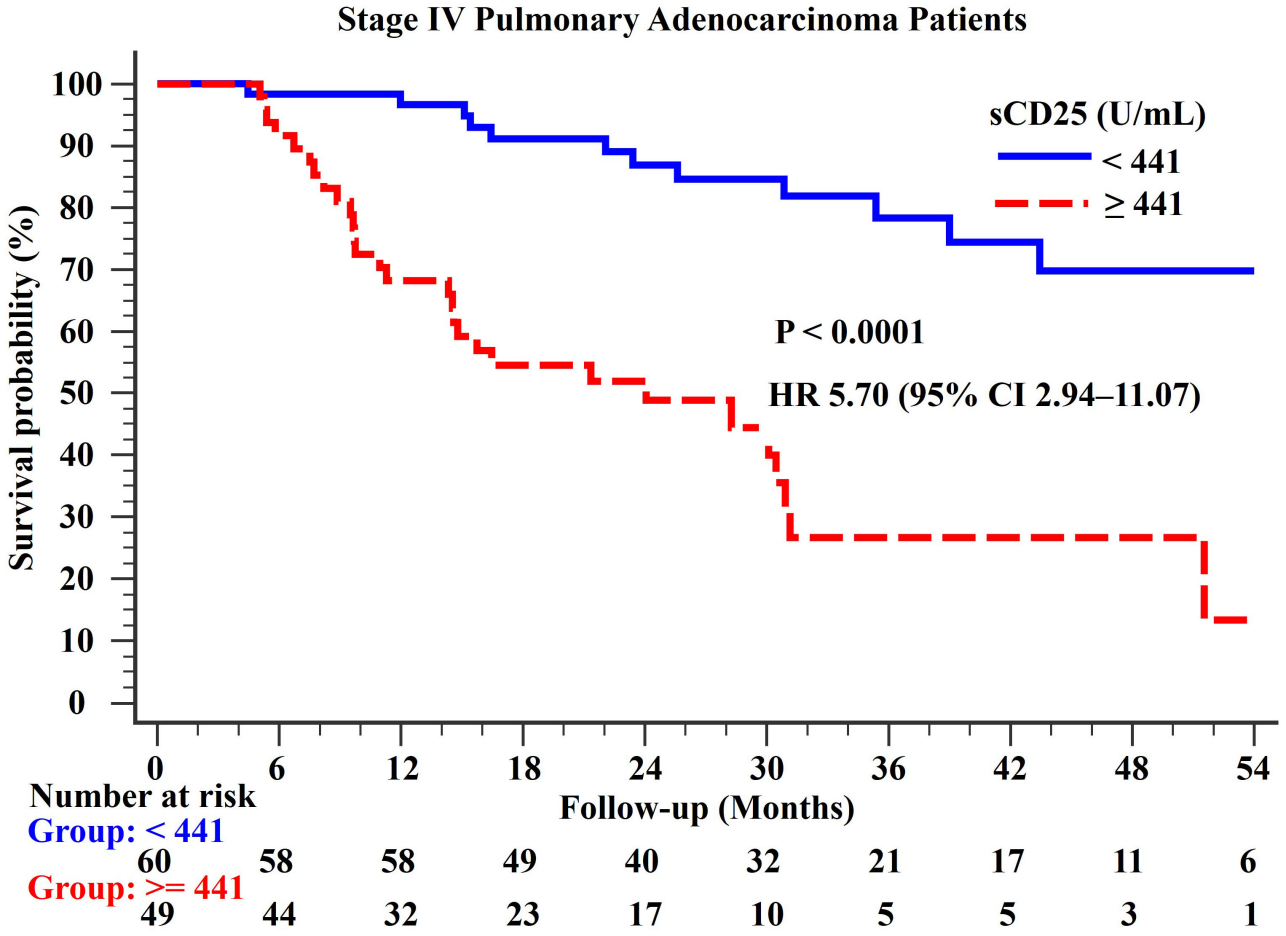
Kaplan–Meier curves comparing overall survival according to baseline sCD25 level. Median overall survival (OS) was not reached in patients with sCD25 <441 U/mL, whereas patients with sCD25 ≥441 U/mL had a median OS of 24.0 months (HR, 5.70; 95% CI, 2.94–11.07; P < .0001). One- and three-year OS rates were 97% and 78% for low sCD25 versus 68% and 27% for high sCD25.

Among 109 patients with evaluable sCD25 data, elevated sCD25 (≥441 U/mL) was strongly associated with inferior overall survival (**Table 1**; **Figure 1**). Median OS was 24.0 months for patients with high sCD25, whereas the median for low-sCD25 patients was not reached. The hazard ratio (HR) for death was 5.70 (95% CI, 2.94–11.07; P <.0001). One- and three-year OS rates were 97% and 78% in the low-sCD25 group compared with 68% and 27% in the high-sCD25 group. However, few events limited power to assess treatment effect.

Propensity score–matched analyses confirmed a robust prognostic effect. In the matched cohort, median OS was 28.3 months for high-sCD25 patients and not reached for low-sCD25 patients (HR, 4.61; 95% CI, 2.01–10.62; P <.001).

Additional performance metrics and classification accuracy for sCD25, including analyses using alternative thresholds, are provided in **Supplementary Tables 4**–**6**.

### Multivariable and propensity score analyses

In multivariable Cox regression, sCD25 ≥441 U/mL remained the only independent baseline prognostic factor (HR, 4.23; 95% CI, 2.01–8.91; P <.001). Ferritin and all other clinical covariates lost statistical significance (**Table 2**). Propensity score matching achieved excellent covariate balance. Results of prespecified models are shown in **Supplementary Figure S2**. Prior to matching, baseline covariates predicted sCD25 status with AUC = 0.774; after matching, discrimination declined to AUC = 0.502, indicating effective removal of imbalance. Detailed baseline characteristics and covariate balance are presented in **Supplementary Table 7**, while survival comparisons between full and matched cohorts are shown in **Supplementary Table 8**.

**Table 2:** Results of multivariable Cox regression show that sCD25 ≥441 versus < 441 is the only independent overall survival predictor.

Variable (comparator)	HR	95% CI	P
Age ≥68 vs <68 y	1.05	0.65–1.70	.76
Male vs female	1.03	0.64–1.67	.94
TNM IVB vs IVA	1.05	0.54–2.03	.90
EGFR mutation vs absent/non-EGFR	0.71	0.36–1.41	.34
BMI <22.1 vs ≥22.1 kg/m²	1.06	0.66–1.72	.89
Ferritin ≥328 vs <328 ng/mL	1.20	0.60–2.38	.63
sCD25 ≥441 vs <441 U/mL	4.23	2.01–8.91	<.001

Hazard ratios are from multivariable Cox regression. HR, hazard ratio; CI, confidence interval; EGFR, epidermal growth factor receptor; BMI, body mass index; sCD25, soluble interleukin-2 receptor.

*Post hoc* power calculations of survival comparisons of sCD25 <441 vs sCD25 ≥ 441 are shown in **Supplementary Table 9**. Sensitivity analyses using alternative matching methods (including 1:1 greedy matching with calliper 0.1) produced consistent results, further supporting robustness (**Supplementary Table 10**).

### Therapy interactions

**Figure 2:**
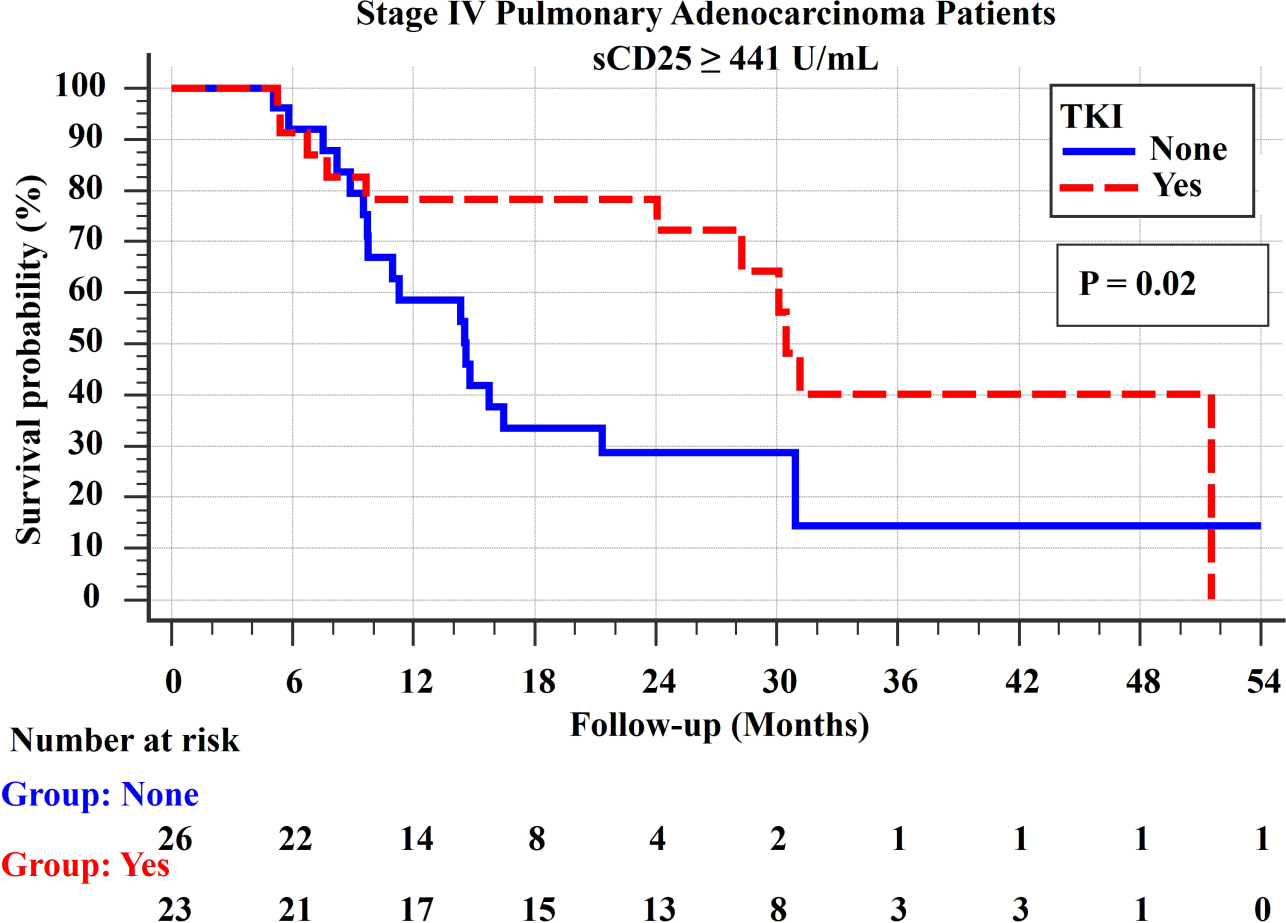
Kaplan–Meier curves showing EGFR–TKI treatment had strong overall survival benefit in patients with sCD25 ≥441 U/mL (HR, 0.41; 95% CI, 0.19–0.88; P = .02). The sCD25 × TKI interaction was significant (P = .02).

**Figure 3:**
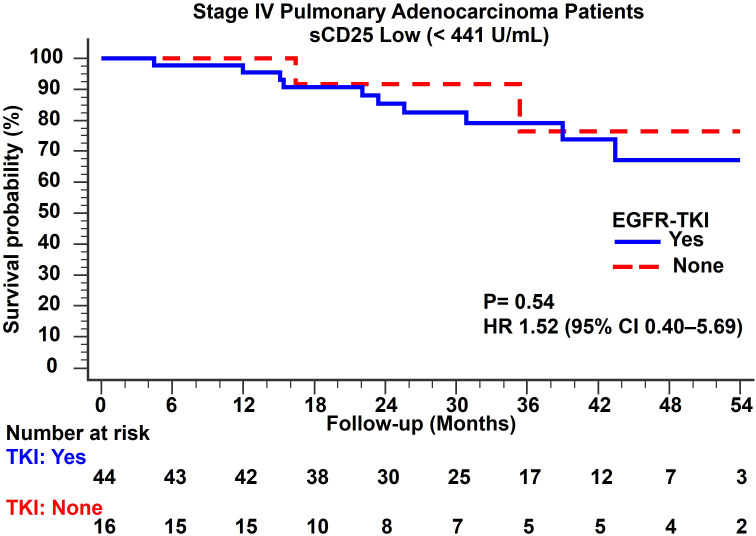
No statistically significant association between EGFR–TKI treatment and overall survival was observed in patients with sCD25 <441 U/mL (HR, 1.52; 95% CI, 0.40–5.69; P = .54). Interpretation is limited by the small number of events.

Analyses were restricted to patients with available EGFR mutation status; given the small number of patients with missing data, this is unlikely to have materially affected the results. A significant interaction was observed between sCD25 and EGFR–TKI therapy (**Table 3**). In patients with high sCD25 (≥441 U/mL), EGFR–TKIs were associated with a marked survival advantage (HR, 0.41; 95% CI, 0.19–0.88; P = .02; **Figure 2**). No statistically significant association was observed among patients with low sCD25 (<441 U/mL; HR, 1.52; 95% CI, 0.40–5.69; P = .54; **Figure 3**). This subgroup had a very favorable prognosis with few events, limiting statistical power to reliably assess treatment effects. The EGFR–TKI × sCD25 interaction was statistically significant (P = .02; **Supplementary Table 11**), suggesting a potential differential treatment effect across biomarker strata; however, this finding should be interpreted with caution given the limited number of events. Baseline characteristics did not differ materially between patients with available and missing sCD25 measurements (**Supplementary Table 12**). No significant interactions were observed for immune checkpoint inhibitors or antiangiogenic therapy (**Table 3**).

**Table 3:** Subgroup survival analyses by sCD25 and therapy.

Therapy type	sCD25 stratum	HR (95% CI)	P
EGFR–TKI	<441 U/mL	1.52 (0.40–5.69)	.54
	≥441 U/mL	0.41 (0.19–0.88)	.02
Antiangiogenic	<441 U/mL	2.14 (0.67–6.80)	.20
	≥441 U/mL	1.00 (0.48–2.09)	.99
Immunotherapy	<441 U/mL	1.74 (0.51–5.98)	.38
	≥441 U/mL	0.88 (0.41–1.89)	.74

HRs (95% CIs) were derived from Cox proportional hazards models with Wald test P values.

### Mutation profile in the low-sCD25 subgroup

Among patients with sCD25 <441 U/mL (n = 60), 61.6% harbored EGFR mutations, 21.6% had no identifiable oncogenic driver, 15.0% had other mutations (including KRAS, ALK, RET, and BRAF), and 1.6% had unknown status. Survival did not differ significantly across molecular subgroups within the low-sCD25 population.

## Discussion

### General

This prospective study demonstrates that elevated baseline soluble CD25 (sCD25; sIL-2Rα) is a powerful prognostic biomarker in stage IV lung adenocarcinoma (LUAD), independently associated with shorter overall survival. Moreover, survival analyses suggest differential benefit from EGFR tyrosine kinase inhibitors (TKIs) across sCD25 strata, supporting a potential treatment-stratifying role for this biomarker. Exploratory analyses of treatment-specific sCD25 thresholds (e.g., for immune checkpoint inhibitors and antiangiogenic therapy) did not identify cutoffs that outperformed the ROC-derived threshold of 441 U/mL, supporting the robustness of this cutoff across treatment modalities.

Notably, patients with low sCD25 had an excellent prognosis with few events, limiting the ability to detect treatment effects in this subgroup. Accordingly, the absence of an observed association should be interpreted with caution. This pattern contrasts with prior NSCLC studies that included heterogeneous stages, mixed histologies, variable ECOG performance status, and substantial proportions of patients with survival <3 months (32, 33). By restricting enrollment to consecutive stage IV LUAD patients with ECOG performance status 0–1 and survival ≥3 months, our analysis reduces these common sources of bias and provides a clearer assessment of biomarker–outcome relationships.

The survival distribution further illustrates the magnitude of sCD25 stratification. Patients with low sCD25 had not yet reached median survival, with the lower quartile at 35 months and the upper quartile not estimable. In contrast, patients with elevated sCD25 demonstrated a compressed survival trajectory, with a median of 15 months and an interquartile range of 10–43 months. These findings indicate that sCD25 captures a clinically meaningful dimension of biological aggressiveness in metastatic ECOG 0–1 LUAD with ≥3 months follow-up. Because the sCD25 cutoff was derived within the same cohort, it is data-driven and potentially subject to overfitting. Although internal validation and consistency across alternative categorizations support its robustness, external validation is required.

### Biological background

The biological basis for these observations may relate to the dual immunologic role of sCD25. Elevated sCD25 reflects systemic inflammation and increased regulatory T-cell activity, contributing to immune suppression and accelerated disease progression (6–9). However, in immunologically “cold” EGFR-mutant tumors, attenuated immune editing may preserve oncogene dependence, thereby enhancing sensitivity to EGFR–TKIs (34, 35). Concurrently, microenvironmental mechanisms—including HGF/MET axis activation and AXL-mediated resistance—may influence therapeutic responses (36–38). These hypotheses remain exploratory and require validation in translational studies.

Other biomarkers performed less robustly in this homogeneous cohort. Ferritin, previously associated with inflammation-driven poor prognosis in multiple malignancies (14–17), lost significance in multivariable analysis. Male sex showed a trend toward inferior outcomes, consistent with emerging data linking Y-chromosome loss to immune dysfunction in cancer biology (39, 40). Additional immunotherapy-related biomarkers—including KEAP1/STK11-related signatures (41, 42), Napsin A–specific clonotypes (43), and IL-6/CRP inflammatory pathways (44)—highlight the complexity of immune–tumor interactions. Within this evolving landscape, sCD25 may represent a clinically accessible biomarker integrating systemic inflammation, immune modulation, and oncogene dependence.

### Therapeutic landscape in EGFR-mutant NSCLC

The therapeutic landscape of EGFR-mutant NSCLC is rapidly evolving toward combination strategies designed to overcome resistance and improve durability of response. Recent phase III evidence demonstrates that dual EGFR–MET targeting with the bispecific antibody amivantamab in combination with lazertinib significantly improves progression-free and overall survival compared with osimertinib, supporting a paradigm shift toward upfront combination therapy (45, 46). Such approaches may be particularly relevant in biologically aggressive subgroups, including patients with elevated systemic immune activation markers such as sCD25.

Recent evidence further highlights the increasing molecular complexity of resistance to EGFR–TKIs, including heterogeneous co-occurring alterations, lineage plasticity, and dynamic clonal evolution under treatment pressure. These findings underscore the importance of integrating biomarker-driven stratification with rational combination therapies (47).

Mechanistic studies provide further insight into resistance biology and therapeutic opportunities. Transcriptional regulation represents one such mechanism, with the transcription factor ZNF263 downregulated in drug-tolerant persister cells and residual tumors. Restoration of ZNF263 enhances sensitivity to EGFR–TKIs, delays resistance development, and suppresses residual disease through epigenetic repression of EGFR signaling (48).

In parallel, metabolic reprogramming contributes to resistance, particularly through accumulation of inosine in TKI-resistant cells. Inosine activates the adenosine A2A receptor and downstream cAMP–PKA–CREB signaling, promoting oxidative phosphorylation, tumor cell survival, and immunosuppressive macrophage polarization. Targeting this axis—either by restoring purine metabolism via purine nucleoside phosphorylase (PNP) or by inhibiting A2A signaling—has been shown to resensitize tumors to EGFR–TKIs in preclinical models (49).

These findings highlight the multifactorial nature of EGFR–TKI resistance, integrating transcriptional, metabolic, and microenvironmental mechanisms. Emerging immunomodulatory targets such as CD24 further contribute to this framework; CD24–Siglec-10 signaling suppresses macrophage-mediated phagocytosis and facilitates immune evasion, and targeting this pathway may enhance responses to EGFR-targeted therapies, particularly in immunologically “cold” tumors (50–52).

Complementary immunosuppressive pathways, including CD73-mediated adenosine signaling, also shape the tumor microenvironment and represent additional therapeutic targets (50, 53). In parallel, TROP2-directed antibody–drug conjugates (ADCs), such as sacituzumab govitecan and datopotamab deruxtecan, have demonstrated encouraging activity in EGFR-mutant and pretreated NSCLC, offering a strategy to bypass canonical resistance mechanisms through targeted cytotoxic delivery (54, 55). Together, these approaches represent an emerging therapeutic paradigm integrating targeted therapy, metabolic modulation, and immune reprogramming.

### Strengths and limitations

Strengths of this study include its prospective design, consecutive enrollment, restriction to ECOG 0–1 stage IV LUAD, exclusion of early deaths that can bias biomarker analyses, and rigorous propensity score methodology. The absence of association between baseline sCD25 and treatment allocation supports its role as a marker of underlying disease biology rather than treatment selection.

Several limitations related to sample size should be acknowledged. Although the overall cohort was sufficient to detect a strong prognostic association between sCD25 and overall survival, the number of events within certain treatment-defined subgroups was limited, particularly among patients with low sCD25 levels who demonstrated favorable survival outcomes. Consequently, subgroup-specific hazard ratio estimates and interaction analyses may be statistically unstable and subject to imprecision, as reflected by relatively wide confidence intervals. These exploratory findings therefore require cautious interpretation and independent external validation.

The limited number of deaths in the low-sCD25 subgroup reduced the ability to reliably detect treatment effects despite prolonged follow-up. Accordingly, the absence of statistically significant associations in some subgroup analyses should not be interpreted as evidence of absence of treatment benefit.

In addition, because the 441 U/mL cutoff was derived and evaluated within the same cohort, there remains a risk of optimism and overfitting despite bootstrap internal validation and consistency across alternative categorizations. External validation in larger multicenter cohorts is therefore necessary before clinical implementation.

Accordingly, the study population was defined *a priori* as patients with stage IV LUAD who survived ≥3 months and were candidates for systemic therapy. This approach introduces survivor selection and limits generalizability but aligns conceptually with landmark and conditional survival analyses (48, 49, 52, 56–59).

## Conclusion

Baseline sCD25 is a clinically meaningful biomarker in stage IV LUAD, independently identifying patients with poor prognosis while suggesting differential benefit from EGFR–TKIs. These findings highlight the value of real-world observational data in identifying clinically actionable biomarkers beyond the constraints of randomized trials.

Future studies should evaluate whether integrating sCD25 with emerging combination strategies—including bispecific antibodies, immunomodulatory targets, and antibody–drug conjugates—can further refine personalized treatment approaches in advanced NSCLC.

## Data Availability

The raw data supporing the conclusions of this article will be made available at reasonable request, from the first corresponding author.
