# Supplementary material for: Real-world evidence of baseline soluble CD25 as a prognostic biomarker and indicator of differential EGFR–TKI benefit in stage IV lung adenocarcinoma
Source: Front Oncol. 2026 Jul 10;16:1810131. doi: 10.3389/fonc.2026.1810131 (PMC13395728; doi:10.3389/fonc.2026.1810131)
Supplement: Supplementary file 1 [file DataSheet1.docx]

# Supplementary file

Supplementary Table S1 shows the Biomarkers and assay details**.** Supplementary Table S2 shows the Standardized Mean Difference (SMD) calculation methods by covariate type. A quality checklist of propensity score analysis is shown in Supplementary Table S3. Supplementary Table S4 shows the prognostic performance metrics for sCD25 and baseline covariates. Supplementary Table S5 demonstrates the survival classification accuracy of sCD25 <441 vs ≥441. Supplementary Table S6 shows the survival accuracy performance using alternative thresholds (402 and 495 U/mL).

Covariate balance was assessed using standardized mean differences (Supplementary Tables S7–S10)

Supplementary Table S7 shows Baseline demographic variables of the study population before and after matching. Post hoc power calculations indicated greater than 95% statistical power for full and matched subgroup comparisons (Supplementary Table S8).

Sensitivity analyses incorporating BMI and diabetes mellitus are shown in Supplementary Table S9. Treatment-by-sCD25 interactions (EGFR–TKIs, immune checkpoint inhibitors, and antiangiogenic therapy) were evaluated. Sensitivity in PSM are provided in Supplementary Table S10. Supplementary Table S11 shows the full interaction mode of treatment-by-sCD25 interactions. Supplementary Tables S12 shows the Baseline characteristics in patients with known and unknown sCD values (Chi-square test).

Of 385 screened patients, 133 consecutive cases met all eligibility criteria. The Strobe flow diagram is shown in Supplement Figure S1. Supplementary Figure S1 shows the Covariate balance using standardized mean differences is visualized in a forest plot (Supplementary Figure S2) (see figures below).

**Supplementary Tables**

**Supplementary Table S1. Biomarkers and assay details.**

| **Biomarker*** | **Equipment or test method** | **Company** | **Normal reference** |
| --- | --- | --- | --- |
| sCD25 | Siemens | Siemens | 223–710 U/mL |
| Ferritin | Roche cobas8000 | Roche | 13.0–150.0 ng/mL |
| VEGF-A | Elisa | Dakawe | 100-300 pg/ml |

*Abbreviations: sCD25, soluble interleukin-2 receptor, VEGF-A, Vascular Endothelial Growth Factor A. VEGF-A measurement discontinued after 2021 due to high assay variability.

Supplementary Table S2. Standardized mean difference (SMD) calculation methods by covariate type.

| Type of covariate | Formula | Description | Explanation |
| --- | --- | --- | --- |
| Continuous covariates | SMD = (Mean₁ - Mean₀) / √[(Var₁ + Var₀) / 2] | Mean₁, Mean₀: group means; Var₁, Var₀: variances | Difference in group means divided by pooled SD (Cohen’s d). |
| Categorical covariates (2 categories) | SMD = (P₁ - P₀) / √[P(1-P)], P = pooled proportion | P₁, P₀: proportions in groups | Difference in proportions divided by SD of pooled proportion. |
| Categorical covariates (>2 categories) | SMD_overall = √[Σ(P₁ᵢ - P₀ᵢ)² / K] | P₁ᵢ, P₀ᵢ: category proportions; K = number of categories | Root mean squared difference in proportions across categories. |

Note: The standardized mean difference (SMD) measures group differences in standard deviation units and is widely used to assess covariate balance in observational studies, particularly with propensity score methods. Threshold for good balance: |SMD| < 0.10. Unlike P values, the SMD is independent of sample size.

Supplementary Table S3. Quality checklist of propensity score analysis.

| Item | Recommendation | Relevant Details | Reported in Section |
| --- | --- | --- | --- |
| Preparation for PS Analysis |  |  |  |
| 1. | Point out scientific background | sCD25 as prognostic/predictive biomarker in stage IV LUAD | Abstract, Introduction |
| 2. | Indicate key study design components | Prospective observational cohort; PS matching to reduce confounding | Methods |
| 3. | State study objectives | Evaluate sCD25 as independent predictor of survival and treatment modifier | Introduction |
| 4. | Describe data sources and variables | Consecutive patients, standardized data collection, biomarker measurements | Methods |
| PS Model Building |  |  |  |
| 5. | Select variables for PS model | Age, sex, TNM stage, EGFR mutation, ferritin (baseline factors only) | Methods |
| 6. | Decide PS estimation method | Logistic regression for propensity estimation | Methods |
| 7. | Evaluate overlap | Adequate common support confirmed across groups | Data Supplement |
| 8. | Present initial balance diagnostics | SMD reported before and after matching | Suppl. Tables S7, S10; Suppl. Fig S2 |
| Application of PS Methods |  |  |  |
| 9. | Specify analysis type | Association estimates (not causal treatment effects) for prognostic biomarker | Not applicable |
| 10. | State PS method used | 1:1 nearest-neighbor matching, caliper 0.1 | Methods |
| 10a) | Detail matching strategy | 1:1 matching ratio with strict caliper | Methods |
| 10b) | Management of extreme weights | Not applicable (matching used, not weighting) | - |
| 10c) | Provide matching details | 64 patients retained from 133 after matching (SMD <0.1, adequate overlap) | Methods, Suppl. Tables |
| 10d) | Describe stratification | Overlap assessment only, not for matching | Data Supplement |
| 11. | Present balance diagnostics post-matching | SMD <0.10 achieved for all covariates | Results, Suppl. Tables S7-S10 |
| 12. | State outcome model | Cox regression, Kaplan-Meier survival analysis | Methods, Results |
| 13. | Perform sensitivity analysis | Stricter matching with additional covariates (n=22) | Suppl. Table S9 |
| 14. | Report effects for all datasets | Full and matched cohort results presented | Results, Tables 1-2, Supplementary Figure S2 |

Verification note:
All section references and page numbers listed in Supplementary Table S2 were cross-checked against the final version of the main manuscript. We confirm that the “Reported in Section” and “Page” columns correspond accurately to the relevant text and pagination in the main manuscript.

Supplementary Table S4. Prognostic performance metrics for sCD25 and baseline covariates.

| Variable | HR (95% CI) | P | Harrell’s C-statistic | Sensitivity % | Specificity % | PPV % | NPV % |
| --- | --- | --- | --- | --- | --- | --- | --- |
| Age ≥68 | 2.30 (1.28–4.11) | .008 | 0.70 | 53 | 61 | 46 | 68 |
| Sex | 1.74 (0.99–3.05) | .05 | 0.65 | 32 | 56 | 31 | 58 |
| EGFR mutations | 0.45 (0.25–0.81) | .008 | 0.63 | 43 | 36 | 30 | 50 |
| sCD25 ≥441 | 5.89 (3.04–11.53) | <0.001 | 0.70 | 69 | 72 | 59 | 78 |
| Ferritin ≥328 | 1.89 (1.08–3.32) | .03 | 0.58 | 61 | 65 | 52 | 73 |

Metrics computed in the 109 patients with sCD25 available.

95% CI: 95% Confidence Interval.

C-statistics from univariable Cox models.

Supplementary Table S5. Survival classification accuracy of sCD25 <441 vs ≥441.

| Outcome | <441 | ≥441 | Total (%) |
| --- | --- | --- | --- |
| Alive at last follow-up | 47 | 20 | 67 (62%) |
| Deceased due to disease | 13 | 29 | 42 (38%) |
| Total (%) | 60 (55%) | 49 (45%) | 109 |

Supplementary Table S6. Performance using alternative thresholds (402 and 495 U/mL).

| sCD25 | <402 | 402–494 | >494 | Total (%) |
| --- | --- | --- | --- | --- |
| Alive at last follow-up | 39 | 9 | 19 | 67 (61.5%) |
| Deceased due to disease | 13 | 1 | 28 | 42 (38.5%) |
| Total (%) | 52 (47.7%) | 10 (9.2%) | 47 (43.1%) | 109 |

Thresholds derived from ROC sensitivity analyses.

Thresholds (402, 495 U/mL) from sensitivity ROC; primary threshold 441 U/mL (Youden).

Supplementary Table S7. Baseline demographic variables of the study population. Analysis limited to 109 patients with complete sCD25 and mutation data (133-28 excluded due to missing data, leaving 105 patients). SMD < 0.10 indicates acceptable balance. n=59/46 pre-PSM and 32/32 post-PSM.

Before PSM

| Variable | <441 (n=59) | ≥441 (n=46) | SMD / P |
| --- | --- | --- | --- |
| Age (years) | 62 ± 11 | 67 ± 9 | 0.51 / 0.016 |
| Sex (M/F) | 27/32 | 35/11 | 0.62 / 0.002 |
| TNM stage | IV–A:28, IV-B:31 | IV–A:15, IV-B:31 | 0.15 / 0.127 |
| EGFR mutations | 38 (64%) | 16 (35%) | -0.59 / 0.003 |
| Ferritin ≥328 | 20 (34%) | 26 (57%) | 0.46 / 0.022 |

After PSM

| Variable | <441 (n=32) | ≥441 (n=32) | SMD / P |
| --- | --- | --- | --- |
| Age (years) | 67 ± 8 | 66 ± 10 | -0.08 / 0.759 |
| Sex (M/F) | 21/11 | 22/10 | 0.07 / 0.790 |
| TNM stage | IV–A:12, IV-B:20 | IV–A:13, IV-B:19 | 0.03 / 0.798 |
| EGFR mutations | 16 (50%) | 15 (47%) | -0.06 / 0.803 |
| Ferritin ≥328 | 17 (53%) | 18 (56%) | 0.06 / 0.802 |

Supplementary Table S8. Kaplan–Meier survival analysis comparing full and matched datasets (<441, ≥441).

| Outcome | Full dataset (n=109) | PSM dataset (n=64) |
| --- | --- | --- |
| Median survival (months) | Not reached (<441), 24.1 (≥441) | Not reached (<441), 28.3 (≥441) |
| Mean survival (months) | 50.1 vs 26.9 | 48.9 vs 30.1 |
| Restricted Mean Survival Time (54 mo) | 46.0 vs 26.7 | 46.9 vs 29.8 |
| Hazard ratio (95% CI) | 5.33 (95% CI, 2.73–10.40). | 4.61 (2.01–10.62) |
| Log-rank P | < 0.001 | < 0.001 |

Abbreviation: 95% CI, 95% confidence interval

Supplementary Table S9. Statistical power analysis of survival comparisons of sCD25 <441 vs sCD25 ≥ 441.

| Parameter | Full dataset | PSM dataset |
| --- | --- | --- |
| Sample sizes | 60 vs 49 | 32 vs 32 |
| Event rates | 21.7% vs 59.2% | 18.8% vs 56.3% |
| Observed HR (95% CI) | 5.92 (3.04–11.53) | 4.61 (2.01–10.62) |
| Statistical power | 99.9% | 96.3% |
| Minimum Detectable HR (80% power) | 2.38 | 3.14 |

Abbreviations: HR, Hazard Ratio; 95% CI: 95% Confidence Interval

Note: Power shown is for the observed effect size and refers to be detectable for HR≈2.0 at 80% power.

Supplementary Table S10. Baseline demographic variables before and after stricter propensity score matching. Sensitivity analysis using stricter matching criteria with body mass index (BMI) and diabetes mellitus as additional matching variables. From the original 133 patients, only 42 had complete data for all variables including BMI and diabetes; propensity score matching further reduced this to 22 patients (11 matched pairs).

Before Stricter PSM

| Variable | <441 (n=26) | ≥441 (n=16) | SMD / P |
| --- | --- | --- | --- |
| Age (years) | 63 ± 10 | 71 ± 7 | 0.94 / 0.017 |
| Sex (M/F) | 13/13 | 13/3 | 0.64 / 0.051 |
| TNM stage | IV–A:13, IV-B:13 | IV–A:5, IV-B:11 | 0.19 / 0.237 |
| EGFR mutations | 19 (73%) | 6 (38%) | -0.72 / 0.026 |
| Ferritin ≥328 | 7 (27%) | 8 (50%) | 0.48 / 0.135 |
| BMI <22.3 | 14 (54%) | 7 (44%) | 0.20 / 0.54 |
| Diabetes | 1 (4%) | 4 (25%) | -0.65 / 0.051 |

After Stricter PSM

| Variable | <441 (n=11) | ≥441 (n=11) | SMD / P |
| --- | --- | --- | --- |
| Age (years) | 68 ± 7 | 68 ± 5 | -0.09 / 0.827 |
| Sex (M/F) | 8/3 | 8/3 | 0.00 / 1.000 |
| TNM stage | IV–A:4, IV-B:7 | IV–A:3, IV-B:8 | 0.09 / 0.648 |
| EGFR mutations | 6 (55%) | 5 (45%) | -0.18 / 0.670 |
| Ferritin ≥328 | 5 (45%) | 6 (55%) | 0.18 / 0.670 |
| BMI <22.3 | 6 (55%) | 6 (55%) | 0.00 / 1.00 |
| Diabetes Mellitus | 1 (9%) | 1 (9%) | 0.00 / 1.00 |

Abbreviations: SMD, standardized mean difference; BMI, body mass index.

Supplementary Table S11. Full model specifications of treatment-by-sCD25 interactions (EGFR–TKIs, immune checkpoint inhibitors, and antiangiogenic therapy).

| Variable | HR (95% CI) | P |
| --- | --- | --- |
| Cox regression without interaction terms | | |
| sCD25 ≥441 U/mL | 4.61 (2.01–10.62) | <.001 |
| EGFR-TKI therapy | 0.47 (0.23–0.95) | .04 |
| Antiangiogenic therapy | 1.39 (0.74–2.63) | .30 |
| Immunotherapy | 0.73 (0.36–1.49) | .39 |
| Cox regression with treatment-by-sCD25 interaction terms | | |
| sCD25 ≥441 U/mL | 37.97 (5.51–261.60) | <.001 |
| EGFR-TKI therapy | 2.36 (0.45–12.55) | .31 |
| Antiangiogenic therapy | 1.87 (0.58–6.06) | .30 |
| Immunotherapy | 2.00 (0.55–7.25) | .29 |
| Interaction terms |  |  |
| TKI × sCD25 | 0.13 (0.02–0.83) | .02 |
| Anti-angiogenic × sCD25 | 0.65 (0.16–2.60) | .54 |
| Immunotherapy × sCD25 | 0.27 (0.06–1.25) | .34 |

Abbreviations: HR, hazard ratio; CI, confidence interval; TKI, tyrosine kinase inhibitor. Main effects in the interaction model represent treatment effects in the low sCD25 group (<441 U/mL). An interaction HR <1.0 indicates greater treatment benefit in the high sCD25 group (≥441 U/mL).

**Supplementary Table S12.** Baseline characteristics in patients with known and unknown sCD values (Chi-square test).

**Characteristic P**

Age (years) 0.54
Sex 0.86
TNM stage 0.11
EGFR mutation 0.36
Type 2 diabetes 0.13

**SUPPLEMENTARY FIGURE LEGENDS**

**
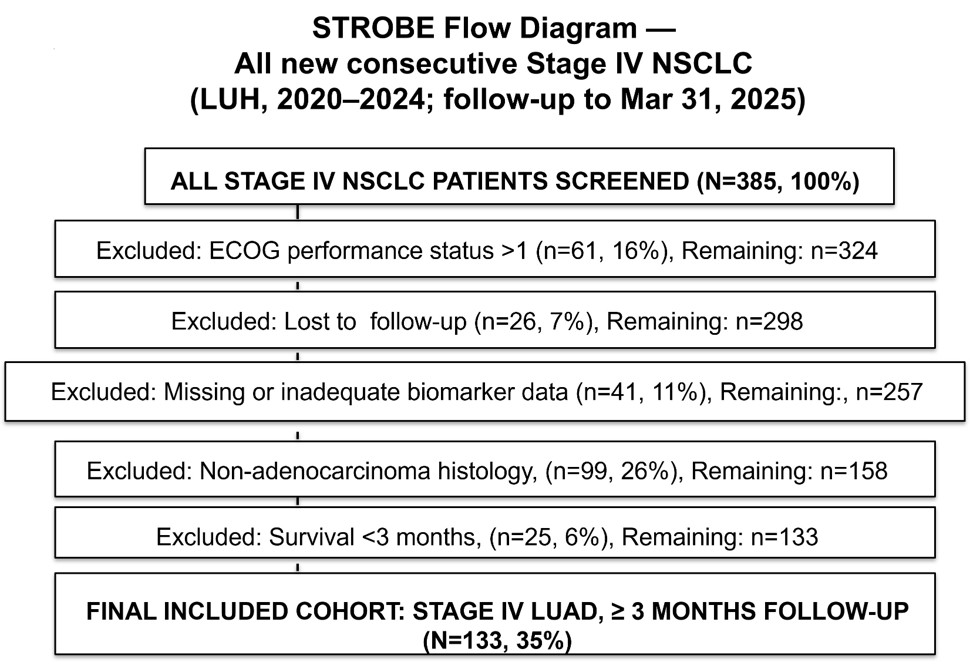
**

**Supplementary Figure S1.** STROBE flow diagram. Consecutive stage IV NSCLC patients at Longhua University Hospital (Jan 2020–Dec 2024; N=385). Excluded: ECOG >1 (n=61), no follow-up (n=26), inadequate biomarkers (n=41), non-adenocarcinoma (n=99), OS <3 months (n=25). Final cohort: stage IV LUAD, ECOG 0–1, adequate baseline labs, ≥3 months follow-up (n=133). Follow-up censored March 31, 2025.

**
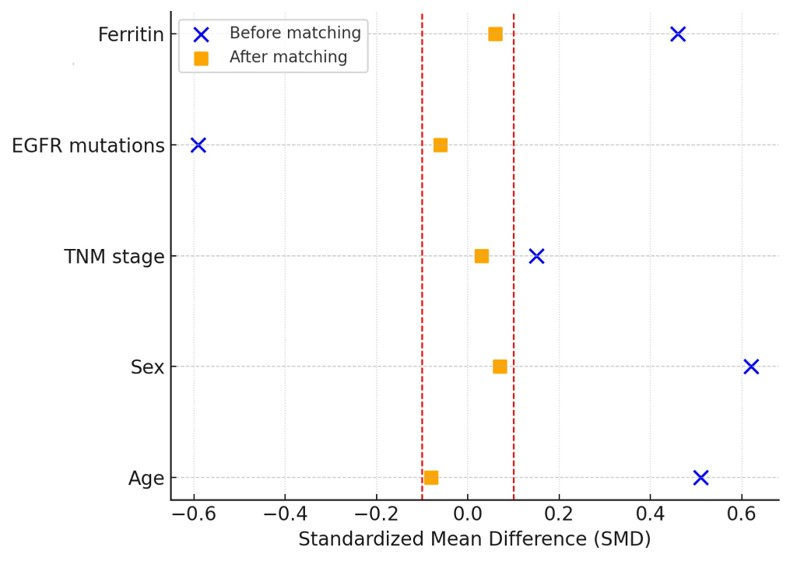
Supplementary Figure S2.** Forest plot showing hazard ratios for overall survival associated with baseline sCD25 across prespecified models in the study cohort (full dataset: n = 109; matched dataset: n = 64). Points indicate hazard ratios and horizontal lines indicate 95% confidence intervals.

**Drug Details**

EGFR–TKIs: osimertinib, icotinib, gefitinib, erlotinib, furmonertinib, dacomitinib, almonertinib, afatinib. Most frequent: osimertinib, icotinib, gefitinib; survival did not differ (P=.42).
ICIs: pembrolizumab, tislelizumab, sintilimab, camrelizumab.
Antiangiogenic: bevacizumab.

**Biomarker Measurements**

sCD25: chemiluminescent immunoassay (Immulite; Siemens; CV <4%).
Ferritin: electrochemiluminescence (COBAS 8000; Roche; CV 4.8%).
VEGF-A: ELISA (Dakewe; CV 25%–30%); discontinued after 2021.

**Abbreviations**
AUC: area under the curve; BMI: body mass index; CV: coefficient of variation; ICI: immune checkpoint inhibitor; LUAD: lung adenocarcinoma; NSCLC: non–small-cell lung cancer; OS: overall survival; PSM: propensity score matching; ROC: receiver operating characteristic; SMD: standardized mean difference; TKI: tyrosine kinase inhibitor; TNM: tumor-node-metastasis staging.
